# Practice Effects and the Lanthony D15

**DOI:** 10.3390/vision10020033

**Published:** 2026-06-10

**Authors:** Joslynn Ho, Jason S. Ng

**Affiliations:** Southern California College of Optometry, Marshall B. Ketchum University, 2575 Yorba Linda Blvd., Fullerton, CA 92831, USA

**Keywords:** colour vision, colour perception, colour vision deficiencies

## Abstract

Purpose: Recent studies have shown that subjects with congenital colour vision deficiency (CVD) can pass the saturated Farnsworth D15 (FD15) through practice. The Lanthony D15 (LD15) uses desaturated colours and is a difficult test even for patients with mild CVD. This study investigated if subjects could improve their performance on the LD15 with intentional practice. Methods: Twenty-one male subjects (mean/SD age = 30.2/8.9 years) with congenital CVD were enrolled in the two-visit study. Their CVD status was evaluated with colour vision book screening, anomaloscope, and FD15 tests. The recruited subjects completed four trials of the LD15 at the baseline visit. Once all data collection was completed at the first visit, it was revealed to the subjects that the correct cap order could be determined by viewing the undersides of the caps. The subjects were given an LD15 test kit to practice at home to improve their performance before returning for a second visit. At the second visit, subjects performed another four trials of the LD15. The colour confusion indices for each trial were recorded for both visits, and the average values were used in paired *t*-tests to determine if significant improvement occurred. Results: A significant decrease in colour confusion index (CCI) of 0.51 (visit 1 mean/SE = 3.75/0.20; visit 2 mean/SE = 3.23/0.24) between the two visits was observed (*p* = 0.01). Further, subjects with deutan deficiency showed a significant (*p* = 0.003) decrease, whereas subjects with protan deficiency did not (*p* = 0.88). Conclusion: Despite an already challenging test, this study showed that subjects with congenital red-green CVD can improve their performance on the Lanthony D15 test. However, this is unlikely to change pass–fail rates when the baseline CCI is high, and further study on this issue is warranted.

## 1. Introduction

Individuals with congenital colour vision deficiency (CVD), acquired through genetics, have one or more of their cone photoreceptors abnormally functioning or absent [1]. These individuals can experience difficulties in performing daily tasks that require them to distinguish a colour of interest [2]. It has been found that people with protanopia or protanomaly, who have reduced sensitivity to long wavelengths, usually have trouble recognizing red traffic signals or brake lights, and are more likely to be involved in road collisions [3]. Beyond interfering with daily life tasks, CVD can also keep affected individuals from entering occupations that require normal colour vision. For example, commercial and military (i.e., drivers and pilots) personnel need to recognize signal light safety and firefighters need to identify colour codes on different equipment or shades of smoke at different stages of a fire [2]. Individuals with CVD who gain entry to occupations involving colour discrimination may have difficulty on the job and/or adversely cause harm to themselves or the community they serve [1]. Therefore, early detection of CVD is essential to career development and work performance for many people.

Amongst clinical colour vision tests, the anomaloscope is considered the gold standard for testing for congenital red-green CVDs; however, it is not usually available in clinical practice due to its expense and complexity [2,3,4,5]. Colour vision book tests, also known as pseudoisochromatic plate tests, such as the Ishihara and the Hardy–Rand–Rittler (HRR) 4th Edition tests, are the most ubiquitous methods for red-green screening in clinical practice [2,3,4]. While these book tests are excellent screening tests because they are easy to administer and have high sensitivity and specificity, they cannot always provide a complete diagnosis (i.e., type and severity) [2,3,4].

Due to the inadequate diagnostic ability of colour vision book tests alone, colour arrangement tests are utilized in conjunction with book tests to broadly classify the type and severity of a CVD. These tests typically comprise a number of coloured caps which patients are asked to arrange in the correct colour order starting with a reference or a pilot cap. Out of the colour arrangement tests, the Farnsworth D15 (FD15) with 16 coloured caps is the most common test in the primary care setting [2,3,4]. Another well-known arrangement test is the Lanthony D15 (LD15). It also has 16 coloured caps; however, the caps use more desaturated colours than those in the FD15, making the LD15 more difficult and more sensitive than the FD15 test, allowing it to assess finer hue discrimination than the FD15 [6,7]. There is, however, limited literature about the clinical significance of potential learning effects on the LD15. Lanthony carried out a research study to determine the effects of repetition on the LD15 on a group of normal trichromats [6]. From the results, he concluded that the subjects showed test improvement after completing three consecutive test administrations and that repeated tests (i.e., trials) are necessary to avoid false positives [6]. Lanthony expressed the view that the best outcome out of three tests would be the most accurate. Similar to Lanthony, Good et al. [7] also recommended administering the LD15 at least three times, but in contrast to Lanthony, Good and colleagues recommended that examiners should average the results of these three tests for each patient, to ensure the accuracy of the LD15. Lanthony and Good et al. used the colour confusion index (CCI) as their scoring method. Both Lanthony and Good et al.’s studies only recruited subjects with normal colour vision, instead of subjects with CVD whose performance might be different. Except for one case report [8], there are no reports evaluating the performance of CVD subjects on the LD15 after practice. Since subjects with severe red-green deficiencies could greatly improve on the FD15 test after a short practice time [9,10], the same possibility should be investigated with the Lanthony D15 more fully. Intentional practice to subvert an FD15 or LD15 test is of concern since both tests have been used to determine suitability for occupational entry. The LD15 is a much harder test, given the desaturated colours, and improvement for patients with CVD may not be possible on the LD15 like it has been shown on the FD15. In this study, we aimed to determine whether patients with congenital red-green CVD could improve their performance on the LD15 with practice.

## 2. Methods

This research was reviewed and approved by an independent ethical review board, the Marshall B. Ketchum University Institutional Review Board (Fullerton, CA, USA; Protocol #21-12), as well as the Graduate Committee of the Master’s in Vision Science Programme (i.e., MS thesis of author J.H.), and conforms with the principles and applicable guidelines for the protection of human subjects in biomedical research (e.g., Declaration of Helsinki). Participants provided written informed consent. Recruitment initiated on 6 December 2021 and concluded on 6 June 2023. Inclusion criteria consisted of subjects who had mild to severe congenital red-green CVD, habitual visual acuity of 0.1 log MAR or better for each eye at far and near, normal ocular and systemic health and a comprehensive eye exam within the past 12 months. Subjects wore their habitual spectacles. Presbyopic patients were required to wear near corrections. Only contact lenses that were clear or had light visibility tints were permitted in the study. Subjects were excluded if they had normal colour vision or any form of acquired CVD. All subjects were compensated for participating at a flat rate. To motivate subjects to improve their performance, extra compensation was offered to subjects who could obtain a perfect LD15 result at the end of the study.

There were two visits for each subject. In the first visit, subjects underwent a case history, visual acuity testing, colour vision screening with book tests, anomaloscope testing, and one FD15 test. If a subject failed the FD15 test, they qualified and were asked to perform the LD15 four times. However, if the subject passed the FD15 test, they performed one trial of the LD15. If that LD15 was perfect, the subject was excluded from further participation due to the floor effect. Otherwise, the subject performed three more trials of the LD15. After visit 1, the subjects were allowed to practice the LD15 at home until they thought they could achieve their best possible result. Then, the subjects returned for a second visit to perform four trials of the LD15 using the specific test set from visit 1 that was kept in the lab, not the one they had taken home.

Visual acuity was measured using standard distance (Precision Vision, Woodstock, IL, USA) and near (Good-Lite, Inc., Elgin, IL, USA) ETDRS charts at 6 m and 40 cm, respectively. Subjects were then screened with colour vision book tests, including the Ishihara 38-plate edition and the HRR (Good-Lite, Inc., Elgin, IL, USA) at a test distance of 75 cm and under the illuminant C equivalent light source (Illuminator with a Verilux Daylight F15T8 fluorescent tube, Good-Lite, Inc., Elgin, IL, USA) at an illumination level of 500 lux. Lastly, the subjects underwent anomaloscope (HMC-Anomaloscope, Oculus, Arlington, WA, USA) and FD15 testing to fully diagnose each subject’s CVD. The FD15 and LD15 were conducted at a test distance of 50 cm, using the same lighting conditions as HRR testing. All colour vision tests were administered binocularly, except for the anomaloscope test. The illuminator light source was found to have stable characteristics between the initiation and termination of data collection (i.e., colour temperature = 6300 K; colour rendering index = 94).

### 2.1. Lanthony D15 Testing

Participants were given a set of 16 coloured caps of the LD15 in a random order. All the undersides of the caps were labelled in order from 1 to 15 except for a starting pilot cap. Each subject completed four trials of the LD15 on the first visit. For each trial, the pilot cap was placed on the left end, and then the subject was instructed to “find the cap that matches closest in colour to the pilot cap and place it next to the pilot cap.” The subject was then instructed to find the next cap in a similar manner until all the coloured caps were used. No viewing of the cap numbers was permitted, but participants were allowed to reorder caps at any time during the test. No feedback was given during or after the test. There was no time limit to perform the LD15 to accommodate any difficulties in handling the caps. The cap arrangement was recorded for each trial.

After completing the first four trials of the LD15 at visit 1, subjects were given a standardized set of loaned equipment consisting of an LD15 test, a standard illuminant C equivalent lamp, and an illuminance metre. They were asked to practice the test at home and to record the amount of practice time on a log. They were also told how to check the caps for their performance (i.e., an order from 1 to 15 was perfect performance). In visit 2, subjects performed another four trials of the LD15. Subjects were not given any advice or strategies on how to improve on purpose. They were instructed to practice for as much time as they could in order to achieve their best possible outcome before returning for a second visit.

The LD15 trials were scored using the colour confusion index (CCI) as the primary outcome measure, which was developed by Bowman and examines the vector distance travelled in colour space for a cap sequence [11,12]. A Total Colour Distance Score (TCDS) is the summation of the colour distances between the sequentially arranged caps, in which the TCDS for errorless performance is 56.4 [11,12]. As a result, quantitative scoring of the LD15 can be determined by calculating a CCI, which is a ratio of the TCDS of an observed result to the TCDS with no errors. The CCI is equal to 1 for a perfect test result and a higher CCI value indicates worse performance [11,12].

Using the method originated by Lanthony [13], error scores were also calculated. These error scores were then compared against the normative data by age reported by Lanthony [13].

Additionally, the metrics developed by Vingrys and King-Smith [14] using colour difference vectors were derived. Among the possible metrics, the confusion angle and the S-index were examined because other metrics such as the C-index are highly correlated to the CCI [15,16]. Thus, they do not add further value and add to the risk of introducing Type 1 errors.

### 2.2. Sample Size

The primary outcome measure used in our study was the CCI value calculated from each LD15 trial. The practical range for a CCI value is approximately 1 to 5. For this study, it was difficult to predict the possible mean differences between two visits. Since the test was more difficult by nature, this could mean that little to no ‘learning’ of the test was possible. However, this situation could have also resulted in a very high CCI at visit 1 with more range to improve, showing ‘learning’. To be conservative, we based the sample size calculation on a mean difference between visit 1 and visit 2 CCI of 1.0 and an assumed standard deviation of the differences equal to 1.5. On these bases, the proposed sample size to achieve an alpha level of 0.05 and a beta level of 0.2 (i.e., power = 0.80) was 20 subjects.

### 2.3. Statistical Analysis

To compare the mean CCI and other metrics of the two visits from the same group of CVD subjects, our data were analyzed using a two-sided paired *t*-test. The four trials of the LD15 from each visit were averaged for each metric. A test of normality was done using Shapiro–Wilk’s test on the distributions to ensure that paired *t*-tests were appropriate to perform. Tests for correlations using the Pearson method between the difference in the CCI numbers, other metrics, and the practice time were also conducted.

## 3. Results

Twenty-one male subjects with congenital red-green CVD were recruited and tested (mean/SD age = 30.2/8.9 years). The habitual visual acuity for both eyes at near had a mean/SD logMAR = −0.05/0.10. The diagnostic type and severity from the anomaloscope and the HRR test are summarized in Table 1.

**Figure 1:**
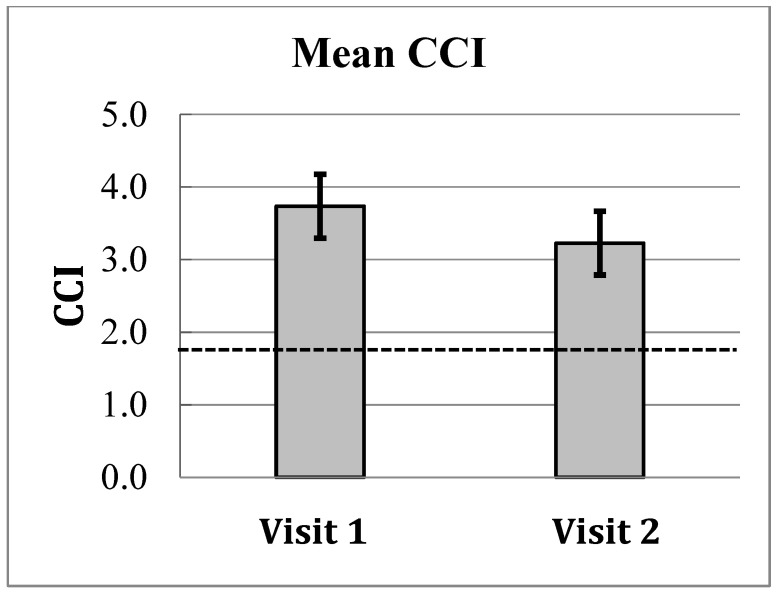
Means of colour confusion indices (CCIs) at visit 1 (left) and visit 2 (right). Error bars are ±2 standard errors. The horizontal line is at 1.75 and represents the upper limit of normal [7].

The distributions of the differences in CCI between the two visits were not significantly different from a normal distribution (*p* = 0.52). The mean/SE of CCI found after visit 1 and visit 2 was 3.75/0.20 and 3.23/0.24, respectively, as shown in Figure 1. The paired *t*-test revealed that the mean CCI from the second visit after practicing was significantly lower than that from the first visit (t = 2.63; *p* = 0.01). The effect size was 0.60. Using this observed effect size in post hoc power calculations showed a sample size of 20 for a one-sided test (appropriate if only examining for improvement) and 25 for a two-sided test (if being very conservative).

**Figure 2:**
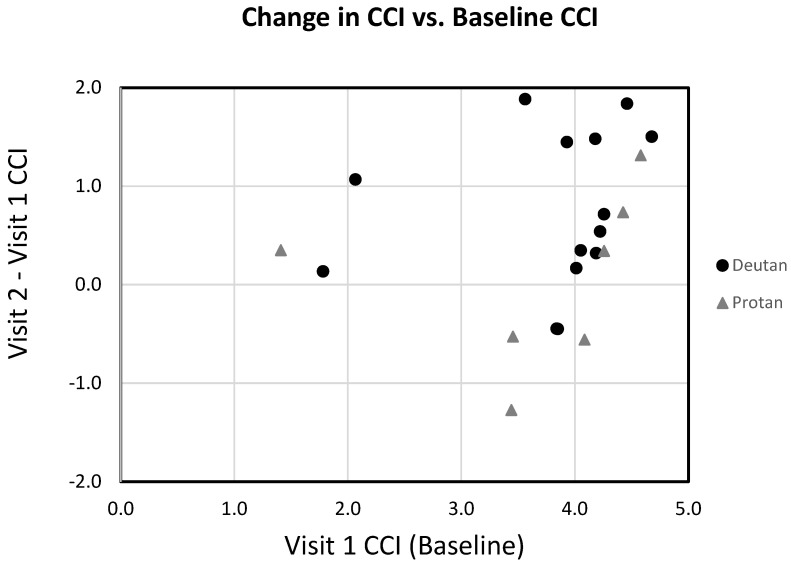
Change in CCI after practice plotted against mean visit 1 CCI. Positive *y*-axis values indicate improvement after practice. Negative *y*-axis values indicate worse performance. Note: Two subjects with deutan deficiency have overlapping points at (3.8, −0.45), and thirteen data points are visualized.

An additional analysis by diagnostic type (i.e., deutan vs. protan) was conducted. It showed that the 14 subjects with deutan deficiency improved their mean CCI from visit 1 to visit 2 by 0.75 (*p* = 0.003), while the 7 subjects with protan deficiency did not improve their mean CCI (0.05 CCI units; *p* = 0.88). This can be visualized in Figure 2. Nearly all of the subjects with deutan deficiency are above the 0.0 line, indicating improvement, while nearly half of the subjects with protan deficiency are below the 0.0 line, indicating worse performance.

Another analysis by dichromacy or anomalous trichromacy was conducted. Due to sample size, only deutan subjects were included in this analysis. Cases of deuteranopia showed significant improvement (*p* = 0.04) and cases of deuteranomaly showed borderline significance (*p* = 0.05). Arguably, if the sole question was improvement (i.e., a one-sided test), then significance is stronger.

Oftentimes, when the Lanthony D15 is used clinically, only one or at most two trials of the test are administered. Analyzing the data for improvement at visit 2 found significant lowering of the CCI in the first and second trials (*p* = 0.03 and *p* = 0.03, respectively).

Beyond CCI, the confusion angle and S-index were also analyzed. The mean confusion angle changed non-significantly (*p* = 0.19) by 3.6 degrees from visit 1 to visit 2 (−1.66 and 1.94, respectively). The mean S-index changed significantly (*p* < 0.001) from visit 1 to visit 2 (4.46 and 3.00, respectively). Similar to the main analysis, when the data were analyzed by diagnostic type, subjects with deutan deficiency followed the same pattern as the whole group’s data: no significant change in angle and a significant change in S-index (4.41 at visit 1 and 2.72 at visit 2, *p* < 0.001). Subjects with protan deficiency did not show any significant changes, very likely due to the small sample size.

The number of perfect cap placements, or tests with a CCI equal to 1, increased from 1 at visit 1 to 7 at visit 2 (i.e., after practice) out of a total of 84 trials, with one subject scoring perfectly on all 4 trials during the second visit despite having no perfect trials during the previous visit. Based on the error scores determined by the method used by Lanthony [13], no subjects would pass the test at visit 1 and two would pass the test at visit 2 (i.e., 0% at visit 1 and 10% at visit 2). Based on the cutoff CCI of 1.75 [7], one subject would pass the test at visit 1 and four subjects would pass at visit 2 (i.e., 5% at visit 1 and 19% at visit 2). The criterion of 1.75 represents the upper limit of all subjects found in the Good et al. study [7].

The total practice time of the patients had a mean/SD of 61.48/54.69 min (range, 11 to 260 min). Pearson tests showed no significant correlations between the difference in the CCIs between two visits and the practice time (*p* = 0.57).

## 4. Discussion

The aim of this study was to determine whether patients with congenital red-green CVD could significantly improve their average CCI with intentional practice. After practicing the LD15, the subjects achieved a significantly lower CCI compared to their pre-practice (i.e., visit 1) CCI (*p* = 0.01). As other known metrics like the C-index are highly correlated with CCI, they were not examined in this study [15,16]. The C-index can also be highly correlated to the S-index on congenital deficiency [17]. This study was not powered to detect differences in pass/fail rates, but the descriptive statistics for pass/fail rates are reported for the reader. There was a general trend towards an increase in pass rates at visit 2.

Based on the sample size calculations as well as a post hoc sample size that could be calculated based on the observed effect size, this study was adequately powered to detect a difference in CCI. Further, analysis of Figure 2 indicates that the effect size was primarily powered by subjects with deutan deficiency as opposed to protan deficiency. It might be thought that subjects with protan deficiency might be able to utilize additional luminance cues that are not available to subjects with deutan deficiency. However, given the desaturated nature of the test, it may be that luminance cues by hue are not as effective as they might be on a test that uses more saturated colours. This may be due to the caps forming a tighter ‘circle’ around the white point on the test as compared to the Farnsworth D15. Perhaps, even more simply, there was not enough statistical power to fully evaluate if subjects with protan deficiency can improve. This can be the subject of a future study.

The confusion angle, which can indicate the type of colour vision deficiency, changed very little in this study and it would not be prudent to make any conjectures based on the observed data. Red-green deficiency typically shows confusion angles between −25 and +25°, while blue-yellow deficiency typically shows confusion angles in the range |70–100| degrees [17]. Based on this, all subjects in this study showed confusion angles consistent with red-green deficiency, except in one instance where subject 20 had a confusion angle in the normal range since they performed perfectly at visit 2. It should be noted that while the confusion angle can be used to separate deutan deficiency and protan deficiency relatively well on the Farnsworth D15, there is a good amount of overlap on the Lanthony D15, making it less useful for that specific test [18].

The S-index is a measure of the symmetry or polarity of the confusions made on the test. A high S-index is indicative of consistent crossings through colour space, whereas a low S-index can indicate random errors or a normal colour vision status [14,15,16]. The statistically significant decrease in the S-index data is likely consistent with a lower number of errors, also reflected in the CCI, but also with the mean at visit 2 being 3.00, there were still consistent errors of a given confusion angle—they did not become random.

Practice time might be thought to correlate with the differences in CCI between the two visits. However, Pearson testing did not show a correlation. One possible explanation is the effectiveness of an individual subject in implementing their own strategies. Subjects need different practice durations to develop their test taking strategies; and there is also likely variability in the efficacy of the strategies that they implemented since no guidance was given.

A prior study tested patients with red-green CVD over 10 trials of the LD15 within a single visit [19]. Their results showed that the CCI was not significantly different across the 10 trials (*p* = 0.18), and no significant learning effect was found in a single visit. The present study showed that the test score can still be affected if enough practice time is allowed for individuals with even severe red-green colour deficiencies to come up with their own strategies. This may impact occupational testing considerations when the test might be used for those purposes, though the potential impact is likely much less than what similar studies with the Farnsworth D15 have shown [9,10].

It was not the purpose of this investigation to determine whether pass–fail rates of the test might be altered by practice. This is an important occupational question that would require a larger sample size. In occupational testing, it is more common to use a coarser measure as an outcome: crossings through the colour circle formed by the caps. Using a coarser measure than the CCI used in this study would not allow smaller differences to be examined. Future investigations should plan their sample sizes around a criterion number of crossings to specifically examine for a change in pass–fail rates.

One study limitation was the uneven distribution in the severity of subjects with colour vision deficiency. Most patients recruited in our study had more severe red-green CVD, which potentially makes observing improvement easier compared to subjects with milder CVD. On the other hand, the more strongly affected a subject’s colour vision, it may be that they have a much harder time showing any improvement. Therefore, a more equal distribution of subjects with mild, moderate and severe colour vision deficiencies could be considered for future studies.

The sample was also imbalanced in the diagnostic type. There were 14 subjects with deutan deficiency and 7 subjects with protan deficiency. While this reflects the greater prevalence of deutan deficiency in the population in general, this limited the study in examining whether practice effects differed among the different diagnostic types. A significant difference in mean CCI was found for subjects with deutan deficiency (*p* = 0.003), but not protan deficiency (*p* = 0.88). The sample size for protan subjects was too small to assess whether such a *p*-value has any merit. On average, subjects with deutan deficiency had a mean improvement/decrease in their CCI by 0.75, while subjects with protan deficiency showed a mean worsening/increase in their CCI by 0.05. Thus, there is at least initial evidence that subjects with protan deficiency have a more difficult time attempting to improve their scores.

A control group was not included in this study because their performance would have likely showed a floor effect on the first trial and thus evidence for improvement would be impossible to discern. Other metrics, such as test time, could conceivably be used, but then this becomes a very different task compared to the colour-deficient subjects. There is little to no evidence that practicing a given colour vision test allows for a transfer of training to another colour vision test, and likely any test-specific gains remain specific to that test. In one report, a subject with dichromacy was able to maintain the ability to perform the Farnsworth D15 perfectly over a year’s time, having never practiced the test during that time. The subject’s anomaloscope data continued to show a full matching range consistent with dichromacy [10]. It is unlikely that subjects who showed learning on the Lanthony D15 test benefited from a form of general learning effects, as compared to test-specific (i.e., individual cap colours) learning. Thus, it is unlikely that utilizing a control group in this study would have impacted the study conclusions.

Lastly, it could be argued that some subjects did not necessarily feel substantially motivated to improve. The motivation given to subjects in this study was extra compensation, but this was a modest amount that was approved by the Institutional Review Board. Indeed, a real-world subject who might be fighting with everything they have to pass an occupational test cannot be truly simulated in a research study. To motivate such a subject to the aforementioned degree may require compensation or other incentive that would likely be considered coercive by any reasonable ethics board. Beyond coercion, this could introduce other conscious or unconscious biases on the examiner or subject side. If one were to take this perspective, then the interpretation of our results would be that the observed difference would be even greater in magnitude.

In conclusion, intentional practice of the LD15 can significantly improve the performance of patients with congenital red-green CVD. Future studies should evaluate whether this improvement rises to clinical significance and potentially alters pass–fail rates that could have occupational testing consequences.

## Figures and Tables

**Table 1:** Summary of testing results.

Subj.	Age	Mean CCI Visit 1	Mean CCI Visit 2	Anom. Range	Type	HRR Severity	Practice Time (min)	Mean Angle Visit 1	Mean Angle Visit 2	Mean S-Index Visit 1	Mean S-Index Visit 2
1	25	4.18	2.70	56	DA	3	52	−10.43	7.28	5.23	2.26
2	30	4.01	3.85	73	D	3	260	−11.98	−13.95	4.77	4.26
3	32	4.26	3.54	73	D	3	26	−11.40	−12.00	5.39	2.56
4	46	4.19	3.87	73	D	3	124	−12.58	−12.90	4.72	3.26
5	22	4.22	3.68	73	D	3	90	−11.68	−12.60	4.88	2.78
6	44	1.78	1.65	73	D	2	86	15.35	17.93	1.77	1.66
7	37	4.58	3.27	73	P	3	65	5.80	6.08	6.12	2.93
8	23	4.43	3.69	73	P	3	26	7.98	7.85	6.32	3.18
9	26	3.46	3.98	73	P	3	11	3.50	8.10	4.15	2.77
10	30	4.05	3.71	73	D	3	35	−10.20	−5.55	4.91	2.69
11	23	1.41	1.06	14	PA	1	13	16.70	−1.90	1.45	1.29
12	40	3.85	4.30	68	DA	3	30	−11.50	−11.63	4.48	2.66
13	28	3.44	4.71	65	PA	3	103	2.70	4.90	3.96	6.39
14	25	4.46	2.62	59	DA	3	51	−6.55	0.13	6.44	2.08
15	26	3.84	4.29	73	D	3	33	−10.40	−8.98	5.32	5.84
16	29	4.09	4.64	73	P	3	57	5.88	2.28	5.59	5.31
17	23	4.68	3.18	73	D	3	16	−8.85	−5.25	6.16	2.83
18	26	3.56	1.68	23	DA	3	39	−8.50	22.03	3.63	1.62
19	54	4.26	3.92	73	P	3	46	−2.33	−2.60	4.43	3.10
20	24	2.07	1.00	32	DA	1	50	24.25	61.40	1.96	1.42
21	22	3.93	2.48	73	D	3	78	−0.73	−9.95	2.06	2.18

CCI = Colour confusion index. Type: D = Deuteranopia; DA = Deuteranomaly; P = Protanopia; PA = Protanomaly. HRR Severity: 1 = mild; 2 = medium; 3 = strong.

## Data Availability

The original contributions presented in this study are included in the article. Further inquiries can be directed to the corresponding author.
